# Appropriate Loads for Peak-Power During Resisted Sprinting on a Non-Motorized Treadmill

**DOI:** 10.2478/hukin-2013-0056

**Published:** 2013-10-08

**Authors:** Matthew J. Andre, Andrew C. Fry, Michael T. Lane

**Affiliations:** 1 Neuromechanics Laboratory, Department of Health, Sport, and Exercise Sciences, University of Kansas, Lawrence, KS, USA.

**Keywords:** resistance, speed, performance, sprint, running

## Abstract

The purpose of this study was to determine the load which allows the highest peak power for resisted sprinting on a non-motorized treadmill and to determine if other variables are related to individual differences. Thirty college students were tested for vertical jump, vertical jump peak and mean power, 10 m sprint, 20 m sprint, leg press 1 RM, leg press 1 RM relative to body weight, leg press 1 RM relative to lean body mass, leg press 1 RM power, and leg press power at 80% of 1 RM. Participants performed eight resisted sprints on a non-motorized treadmill, with increasing relative loads expressed as percent of body weight. Sprint peak power was measured for each load. Pearson correlations were used to determine if relationships between the sprint peak power load and the other variables were significant. The sprint peak power load had a mode of 35% with 73% of all participants having a relative sprint peak power load between 25–35%. Significant correlations occurred between sprint peak power load and body weight, lean body mass, vertical jump peak and mean power, leg press 1 RM, leg press 1 RM relative to lean body mass, leg press 1 RM power, and leg press power at 80% of 1 RM (r = 0.44, 0.43, 0.39, 0.37, 0.47, 0.39, 0.46, and 0.47, respectively). Larger, stronger, more powerful athletes produced peak power at a higher relative load during resisted sprinting on a non-motorized treadmill.

## Introduction

Resisted sprinting (RS) has been shown to improve sprint performance, particularly acceleration over distances less than 10 m, which would be valuable for many sports (Behrens and Simonson, 2011; Hrysomallis, 2012; Ross et al., 2009). While there are several different modes of RS, one could use a non-motorized treadmill with adjustable levels of resistance. Most RS studies use a specific load for all participants (e.g. 7% of bodyweight), therefore, it is unclear whether or not different athletes should use heavier or lighter loads during RS. Alcaraz et al. (2008) concluded that, during RS, high relative loads should be used to elicit the desired response, however, if the load is too heavy, then it may negatively impact sprint technique. The authors (Alcaraz et al., 2008) used sled, parachutes, and weight belts, but they only used one load per device. Therefore, comparisons cannot be made for each device with greater or lesser resistance from this study.

One non-motorized treadmill has been demonstrated to accurately assess horizontal peak power during a sprint (Tong et al., 2001). This same treadmill allows users to modify the amount of resistance to perform RS, thus allowing one to monitor horizontal sprint power during RS. Since power is important for sports performance, it may be beneficial to use the RS load specific to each athlete that helps the individual attain their highest peak power during the sprint.

Previous studies (Alcaraz et al., 2008; Jandacka and Beremlijski, 2011; Jaskolski et al., 1996; Sweeney et al., 2010) have determined that athletes achieve peak power at different relative loads from non-athletes and at different relative loads during different exercises. Jandacka and Beremlijski (2011) found that the optimal load for peak power in a bench press exercise in highly-trained soccer players was 40% 1 RM, although this is an upper-body exercise which did not require the athlete to move the remainder of their body mass. Conversely, Sweeney et al. (2010) determined that resisted sprints on a non-motorized treadmill at 15% of body weight allowed subjects to reach peak power within 3–5 s, although that still does not indicate the appropriate relative load for achieving the highest possible peak power. Additionally, heavy resistance training has been shown to increase power in trained athletes, which may indicate that increases in strength may lead to a need for greater resistance to achieve peak power during RS (Hermassi et al., 2011). This research indicates the need to evaluate the optimal relative load for peak power during resisted sprints on a non-motorized treadmill.

One study (Jaskolski et al., 1996) used a non-motorized treadmill with similar mechanisms and similar power-deriving abilities as the one used in Tong et al.’s (2001) study. The authors (Jaskolski et al., 1996) used 5 s resisted sprints at 5, 8, 10, 13, 15, and 20% of body weight, and determined that the optimal ranges for measuring power on that particular treadmill ranged between 10–15%. Jaskolski et al. (1996) concluded that body mass and athletic ability may affect the optimal power load for resisted sprinting on a non-motorized treadmill. More research is needed to help determine which factors are most related to the appropriate load for achieving the highest peak power during resisted sprinting on a non-motorized treadmill. Therefore, the purpose of this study was to determine the relative load for resisted sprinting on a non-motorized treadmill which allows each athlete to achieve their highest peak power and to determine if other variables are related to individual differences.

## Material and Methods

### Participants

Healthy, male, physically-active college students (mean±SD; n = 30, age = 22±2.4 yrs, body height = 178.6±6.6 cm, body mass = 80.5±13.0 kg) were recruited as voluntary participants in the study. Subjects were physically-active, but not currently competitive athletes, and demonstrated a broad range of physical abilities. All participants signed an Informed Consent document. Approval from the University Human Subjects Committee was received prior to recruitment.

### Procedures

This descriptive study involved observational research used to determine a method for finding the appropriate relative peak power load during a resisted sprint. Thirty college students reported for two sessions with different performance variables tested at each session. Pearson correlation coefficients were used to observe relationships between the appropriate peak power load and other performance variables.

Participants took part in one informational session and two performance-testing sessions. During the informational session, informed consent was obtained and descriptive information was collected. Percent of body fat was determined using a 3-site skinfold caliper test (Lohman, 1981).

During the second session, which was the first performance-testing session, participants were tested for vertical jump (VJ), vertical jump peak power (VJPP), vertical jump mean power (VJMP), 10 m sprint (10S), and 20 m sprint times (20S). First, participants performed a dynamic warm-up, consisting of a submaximal 100 m run, arm circles, leg swings, skipping exercises, and submaximal jumps. Participants then performed five vertical jumps for height, which were measured by a Vertec Jump Measurement System (JumpUSA, Sunnyvale, CA, USA), with one minute of rest between jumps. The highest jump was used for analysis. VJPP and VJMP were determined using equations from Johnson and Bahamonde (1996).

After VJ testing was complete, participants rested for 5 min before performing 3 maximal 20 m sprints with 3 min of rest between sprints. Sprint times were recorded using a wireless TC-System (Brower Timing Systems, Draper, Utah, USA). Timing gates were set at 10 and 20 m so that both distances could be recorded simultaneously. Participants used a standing 2-point start position. The timing clock started when the subjects’ rear foot left the ground. All performance testing for session 2 was conducted in a large, open gymnasium with wooden floors.

Participants attended a third and final session, during which they performed eight resisted sprints on a non-motorized treadmill (Force 3.0, Woodway, Waukesha, WI, USA). Similar to session 2, this session was preceded by a dynamic warm-up involving calisthenics, submaximal walking, and submaximal jogging on the treadmill. Chia and Lim (2008) determined that peak power elicited during repeated sprints on non-motorized treadmills can be impacted by a rest period, and indicated that it is essential to use a minimum of 2 min rest between efforts to maintain a consistent peak power measurement. Therefore, 3 min of seated rest was given between sprints. Resisted sprints were performed with non-randomized increasing relative loads expressed as percent of body weight: 5, 10, 15, 20, 25, 30, 35, and 40 percent of body weight. Sprint peak power (SPP) was measured for each load to help determine which relative load elicited each participant’s greatest SPP.

On the rear shaft of the treadmill there is a speed sensor that directly measures the distance. The sensor is a digital encoder and the resolution is 2 cm per pulse. Pacer software digitally filters (at selected cut-off frequency) the distance/time data and then differentiates using the finite difference technique to produce velocity data. Pacer software digitally filters (at selected cut-off frequency) the distance/time data and then double differentiates using the finite difference technique to produce acceleration data. Pacer software calculates the product of the instantaneous velocity and horizontal force to determine the instantaneous power. Horizontal force is directly measured from the load cell connected to the user’s waist tether. Vertical force is directly measured from the 4 load cells mounted under the running belt. An older version of the Woodway has previously been validated to be able to assess power (Lakomy, 1984).

After SPP testing was complete, participants were given 5 min of seated rest before being tested for leg press 1 RM (LPMAX), leg press 1 RM relative to body weight (RELBW), leg press 1 RM relative to lean body mass (RELLBM), leg press 1 RM power (MAXPOW), and leg press power at 80% of 1 RM (LP80POW) using an Air300 Leg Press (Keiser, Fresno, CA, USA). The Air300 is a pneumatic leg press, meaning that resistance is provided by air pressure. Additionally, the Air300 can calculate peak power. Participants performed submaximal leg presses with 2 min between attempts until they achieved a 1 RM. After 2 min of rest, participants performed 3 maximal-velocity repetitions at 80% of 1 RM. Leg press testing was administered after the resisted sprints to avoid pre-fatiguing the participants. All performance testing for session 3 was conducted in the laboratory.

### Statistical Analysis

Pearson correlation coefficients were used to determine if relationships between the SPP load and the other variables were statistically-significant. A single sample chi-square test was used to determine if differences in frequency of SPP load were statistically-significant. Significance was set a priori (α = .05). Statistical analyses were conducted using IBM SPSS Statistics 20 software.

## Results

Performance testing results are reported in Table 1. The load at which participants achieved peak power had a mode of 35%. Results of the chi-square test are presented in Table 2. Pearson correlation coefficients, which were calculated to assess relationships between participants’ relative sprint peak power load and the remaining variables, are reported in Table 3.

## Discussion

Larger, stronger, more powerful athletes produce peak power at a higher relative load than smaller, weaker, less powerful ones during resisted sprinting on a non-motorized treadmill. This is similar to Chia and Lim (2008), who determined that lower-body mass was positively related to power output during repeated sprints on a non-motorized treadmill. However, the results of this study differ from those of Sweeney et al. (2010), who assessed sprint peak power with American football players and recreationally-trained athletes during resisted sprints on the same non-motorized treadmill as the one used in our study. They suggested that 15% of body weight was the heaviest resistance that allowed participants to achieve peak power within 3–5 s (Sweeney et al., 2010). Additionally, Jaskolski et al. (1996) concluded that the optimal resistance for peak power while sprinting on a different treadmill, which was mechanistically similar to the one used in the present study, was between 10–15 percent of body weight.

During the current study, 73% of participants achieved peak power between 25–35 percent of body weight. Therefore, if the training goal is to achieve peak power during a resisted sprint on a non-motorized treadmill, the load may need to be heavier for some individuals than what has been previously suggested.

The significant relationships between the SPP load and body weight, LBM, VJPP, VJMP, LPMAX, RELLBM, MAXPOW, and LP80POW suggest that there are variables that may help predict at what relative load an athlete will achieve SPP during a resisted sprint on a non-motorized treadmill. For example, since body weight and lower body strength are significantly related to the relative SPP load, a football lineman who is larger and stronger than an endurance athlete will likely need a heavier relative load to achieve SPP than the smaller, weaker athlete. Additionally, increases in strength leading to increases in power via resistance training similar to what was found by Hermassi et al. (2011) may mean that over an athlete’s career, the relative load needed to achieve peak power during RS may increase as the athlete becomes stronger and more powerful. When two athletes jump the same height, the athlete who weighs the most will produce the most power during the jump. During this study, VJPP and VJMP were significantly related to SPP while VJ was not, which also indicates that body weight plays an important role in choosing the relative SPP load. Another important caveat in the discussion of these variables is the relationship between body weight and LPMAX. Body weight and leg press 1 RM had a significant positive correlation (*r* = .87; *P<*.001). Since these variables are highly related, and both variables are related to the relative SPP load, one can assume that simply assessing an athlete’s weight or lower body strength should give an indication of what their relative SPP load should be. In addition, increases in athletes’ lower-body strength which lead to increases in power, as seen in Hermassi et al.’s study (2011), may lead to an increase in the SPP load needed to elicit peak power.

In the present study, five participants weighed more than 90 kg. Of those five participants, four had a relative SPP load of 30% body weight or greater, while the remaining participant had a relative SPP load of 25%. Considering this information, relative SPP loads for athletes weighing more than 90 kg should sometimes be 25% or greater. We attempted to use multiple regressions to determine whether or not we could devise an equation to predict the SPP load based on bodyweight and other variables, but were unable to find a statistically-significant combination of variables. Additionally, a discriminant analysis was used in an attempt to predict the optimal SPP load, however, the statistical software determined that none of the variables were qualified for that analysis. It is possible that this may be improved by using samples with different physical abilities, particularly athletes, or a larger sample size may be necessary.

While variables have been identified to help coaches select appropriate loads for resisted sprinting on a non-motorized treadmill, it is not yet known exactly how resisted sprinting on a non-motorized treadmill should be incorporated into a strength and conditioning program. One training program which was successful in improving sprint speed by utilizing resisted sprinting on a non-motorized treadmill involved weekly changes in load, varying from 0–25 percent of body weight (Ross et al., 2009). It may be beneficial to also include heavier relative loads during some training sessions for larger, stronger athletes, as part of their program.

The results of this study do not confirm or deny the usefulness of performing resisted sprints on a non-motorized treadmill with relative loads that elicit peak power. Rather, this study provides suggestions for determining relative SPP loads, for those who may choose to incorporate this type of training into a well-rounded program. Future research should determine these relationships in different groups of athletes, women, and with different age groups.

Additionally, there are several weaknesses of this study which should be addressed in future research. For example, it is difficult to translate the results of this study to athletic populations. Therefore, future research should include athletes from sports that have an emphasis on power and acceleration, such as football, baseball, and soccer. Finally, an alternative method of performance testing may be appropriate. In this study, jumps and sprints were grouped into one session, while resisted sprints and leg press assessments were grouped into another session. It is possible that fatigue from the resisted sprints may have impacted the leg press assessment. While the authors of this study felt that this was an appropriate testing approach, it may be optimal to have an additional, separate testing session for the leg press assessment.

Jaskolski et al. (1996) discussed the need for repeating treadmill testing, as they found that power improved when the test was repeated on a different day. They suggested that this may be due to improvements in technique from practicing using the treadmill. Similar to the current study, Jaskolski et al. (1996) found that participants struggled to maintain balance, especially at the lighter loads, which may affect the ability to give maximal effort. Therefore, future studies should include multiple familiarization sessions, and look at changes in peak power over multiple practice sessions.

Also, for non-athletes with different athletic abilities, it is possible that fatigue incurred during the first few resisted sprints may have affected performance on subsequent loads. The results of Cooke and Whitacre (1997) indicated that, during repeated maximal cycle sprints, fatigue set in much sooner and had a greater impact on some subjects as compared to others. Additionally, Chia and Lim (2008) found that fatigue from previous sprints could impact peak power on subsequent sprints on a non-motorized treadmill. Therefore, effort should be made to reduce the affects of fatigue during repeated testing.

When choosing the optimal load for peak power during resisted sprinting on a non-motorized treadmill, coaches, researchers, and athletes should consider the athlete’s body-weight and lower body strength and power. Larger, stronger, more powerful athletes should incorporate heavier relative loads than smaller, weaker, less powerful athletes, when attempting to achieve peak power during a resisted sprint on a non-motorized treadmill.

## Figures and Tables

**Table 1:** Performance Testing Results (mean±SD)

Vertical Jump Height (cm)	65.3±8.0
Vertical Jump Peak Power (W/kg)	5945.4±944.7
Vertical Jump Mean Power (W/kg)	2923.0±497.0
10 meter Sprint (s)	1.91±0.09
20 meter Sprint (s)	3.21±0.13
Leg Press 1 RM (kg)	237.2±51.9
Leg Press 1 RM (relative to BW)	2.9±0.4
Leg Press 1 RM (relative to LBM)	3.5±0.5
Leg Press Power at 1 RM (W)	605.1±162.3
Leg Press Power at 80% 1 RM (W)	1000.3±220.9

**Table 2:** Chi-Square Analysis

Load	5%	10%	15%	20%	25%	30%	35%	40%
Expected *n*	4.3	4.3	4.3	4.3	4.3	4.3	4.3	4.3
Observed *n*	2	1	0	4	7	7	8	1

χ^2^= 12.93 (χ^2 crit^=12.59); P = .044

**Table 3:** Pearson Correlation Coefficients

	Peak Power Load	*P*
Body Weight	.44^*^	.016
Lean Body Mass	.43^*^	.017
Vertical Jump Height	.10	.607
Vertical Jump Peak Power	.39^*^	.032
Vertical Jump Mean Power	.37^*^	.043
10 meter Sprint	−.20	.292
20 meter Sprint	−.20	.289
Leg Press 1 RM	.47^**^	.010
Leg Press 1 RM (relative to BW)	.25	.192
Leg Press 1 RM (relative to LBM)	.39^*^	.036
Leg Press Power at 1 RM	.46^*^	.011
Leg Press Power at 80% 1 RM	.47^**^	.009

* p<0.05,

** p<0.01

## References

[b1-jhk-38-161] Alcaraz PE, Palao JM, Elvira JL, Linthorne NP (2008). Effects of three types of resisted sprint training devices on the kinematics of sprinting at maximum velocity. J Strength Cond Res.

[b2-jhk-38-161] Behrens MJ, Simonson SR (2011). A comparison of the various methods used to enhance sprint speed. Strength Cond J.

[b3-jhk-38-161] Chia M, Lim J (2008). Effect of fatigue on mass exponents and power in all-out intensity repeated sprints on a non-motorized treadmill in sedentary adults. Asian J Exerc Sports Sci.

[b4-jhk-38-161] Cooke WH, Whitacre CA (1997). Measuring fatigue relative to peak power output during high-intensity cycle sprinting. Res Q Exerc Sport.

[b5-jhk-38-161] Hermassi S, Chelly MS, Tabka Z, Shephard RJ, Chamari K (2011). Effects of 8-Week in-Season Upper and Lower Limb Heavy Resistance Training on The Peak Power, Throwing Velocity, and Sprint Performance of Elite Male Handball Players. J Strength Cond Res.

[b6-jhk-38-161] Hrysomallis C (2012). The effectiveness of resisted movement training on sprinting and jumping performance. J Strength Cond Res.

[b7-jhk-38-161] Jandacka D, Beremlijski P (2011). Determination of strength exercise intensities based on the load-power-velocity relationship. J Hum Kin.

[b8-jhk-38-161] Jaskolski A, Veenstra B, Goossens P, Jaskolska A, Skinner JS (1996). Optimal resistance for maximal power during treadmill sprinting. Res Sports Med.

[b9-jhk-38-161] Johnson DL, Bahamonde R (1996). Power output estimate in university athletes. J Strength Cond Res.

[b10-jhk-38-161] Lakomy HKA (1984). An ergometer for measuring the power generated during sprinting. J Physiol.

[b11-jhk-38-161] Lohman TG (1981). Skinfold and body density and their relationship to body fatness: a review. Hum Biol.

[b12-jhk-38-161] Ross RA, Ratamess NA, Hoffman JR, Faigenbaum AD, Kang J, Chilakos A (2009). The effects of treadmill sprint training and resistance training on maximal running velocity and power. J Strength Cond Res.

[b13-jhk-38-161] Sweeney KM, Wright GA, Brice AG, Doberstein ST (2010). The effect of b-alanine supplementation on power performance during repeated sprint activity. J Strength Cond Res.

[b14-jhk-38-161] Tong RJ, Bell W, Ball G, Winter EM (2001). Reliability of power output measurements during repeated treadmill sprinting in rugby players. J Sport Sci.

